# Biomonitoring of Mercury and Lead Levels in the Blood of Children Living near a Tropical River Impacted by Artisanal and Small-Scale Gold Mining in Colombia

**DOI:** 10.3390/toxics12100744

**Published:** 2024-10-13

**Authors:** Eurípides Palacios-Valoyes, Manuel H. Salas-Moreno, José L. Marrugo-Negrete

**Affiliations:** 1 Biosistematic Research Group, Biology Department, Faculty of Naturals Sciences, Universidad Tecnológica Del Chocó, Quibdó 270002, Colombia; euripides.palaciosvaloyes@yahoo.com (E.P.-V.); d-manuel.salas@utch.edu.co (M.H.S.-M.); 2 Faculty of Basic Sciences, Universidad de Córdoba, Carrera 6 No. 76-103, Montería 230002, Colombia

**Keywords:** blood, heavy metals, child population, Atrato river basin, gold mining

## Abstract

(1) Background: Mercury and lead contamination resulting from various anthropogenic activities represents a global environmental problem and a considerable risk to the health of the human population. (2) Methods: The objective of this research was to evaluate the concentrations of mercury (Hg) and Lead (Pb) in the blood of the child population in the municipalities in the Atrato River basin using a direct Hg analyzer and graphite furnace atomic absorption spectrometry. (3) Results: In total, 171 children (5–14 years of age) were taken into account, and 18.71% (32) of the children had concentrations of Hg and Pb above the permissible values established by the WHO. In the municipality of UN, 19 children had blood Hg concentrations between 5.29 and 17.71 μg/L. In CA, two children had concentrations of 5.03 and 8.43 μg/L, separately. In the case of Pb, seven children showed concentrations between 3.60 and 4.83 μg/dL in the municipality of RQ, three in UN (3.59, 3.61, and 4.60 μg/dL), and one in Carmen de Atrato (5.47 μg/dL). (4) Conclusions: The levels of Hg and Pb in the blood of children living in the riparian areas of the Atrato River basin are related to gold mining activities in the basin and the consumption of contaminated fish.

## 1. Introduction

Gold mining is an important economic activity in many countries; however, it is also an activity that generates global concern as a source of pollution. During this activity, large amounts of residual substances with heavy metals are used, produced, and discharged, such as arsenic (As), cadmium (Cd), mercury (Hg), and lead (Pb), among others (Rahman and Singh, 2019). These metals contaminate environmental systems and have the peculiarity of being toxic, generating harmful effects on the health of the human population [1]. Human exposure to metals occurs when these metals enter the food chain due to their presence in soil, water, and dust particles, and absorption mainly occurs through food and water consumption. High concentrations of heavy metals have health consequences that can be mild to severe, resulting in conditions in people, like other organisms, ranging from mutations in cellular DNA to damage to the nervous, digestive, reproductive, kidney, and immune systems. Similarly, they produce lung inflammation and the appearance of resistant bacteria [2,3].

Recent reports from recent years indicate that exposure to Pb in middle- and low-income countries has resulted in the deaths of 5,545,000 adults from cardiovascular disease and another 729 million intelligence quotient points lost, along with affecting 765 million children under 5 years of age [4]. Furthermore, exposure to low levels of certain heavy metals can have a cumulative effect over the years [5]. The problem of Pb in the child population is so marked that the Lead Exposure Prevention and Advisory Committee (LEPAC) suggested to the Centers for Disease Control and Prevention (CDC) in 2019 the reduction of the reference value of Pb in blood from 5 μg/dL to 3.5 μg/dL [6]. This heavy metal is associated with gold mining through the extraction process; transportation of gold ore for threshing, washing, and crushing; storage of the processed material; and mining waste [7]. Pb has very marked harmful effects on human health, which are exacerbated in children, generating learning delays, neurocognitive and behavioral disorders, cardiovascular diseases, respiratory problems, and cancer [7,8]. Hg is mainly obtained from atmospheric deposition, and the main organic form of mercury in the food chain is methylmercury (MeHg), which is mainly transformed from inorganic mercury by microorganisms [9]. Taking into account that much of the Hg in foods such as fish is in its MeHg form, exposure to this contaminant could cause learning and behavioral disorders, cardiovascular diseases, and reproductive damage [10,11,12,13]. Children are especially susceptible to MeHg, due to the greater absorption capacity given the greater food intake per amount of body mass, lower rate of detoxification, and the ongoing development of their nervous systems. Gold mining in Colombia poses a direct threat of exposure to Hg and other heavy metals. According to the calculations of the 2013 world assessment on Hg, around three million women and children work in this sector [14]. Colombia is the third largest emitting source of Hg after China and Indonesia [15,16]. Among the waste produced during this type of operation are Pb and Hg. Both metals are generated by the removal of minerals from the soil, drainage of mounds of disturbed soil, water from mining processes, and their waste. In addition, elemental Hg is used to amalgamate the gold, which generates greater amounts of contamination. Elemental mercury is inhaled into the lungs, crosses the blood–alveolar gas barrier, accumulating in different tissues, including the kidneys and brain, causing long-term physiological and neurological damage [17,18]. Additionally, mercury exposure affects important functions of neuropsychological performance in children and adolescents [19].

The department of Chocó is an area with a mining vocation, with low quality of life rates and a series of problems generated mainly by the exploitation of gold in the open pit with backhoes, in jungle areas with dredgers, and in rivers with dredgers or dragoons. The associated problems include deforestation, sedimentation of the Atrato river basin, and contamination with highly toxic heavy metals. The Atrato basin has been ancestrally inhabited by Afro-Colombian and indigenous communities. Based on the Judgment T-622 of 2016 [20] and the Minamata agreement of 2013 [14], the aim is to adopt measures that promote the protection, conservation, maintenance, and restoration of the basin, as well as the protection of the rights to life, health, territory, food security, and culture of the communities that live in the river basin. Reports show 0.07–6.47 ug/g mercury levels in the hair in the inhabitants of Paimadó (Río Quito population center) [21]. In addition, levels of 0.10 µg/g have been reported in sediments of the Río Quito, 0.12 µg/g at the mouth of the Atrato River basin, and 0.09 and 0.09 µg/g for the Tarena and Tigre rivers, respectively (Unguía population center) [22]. In addition, the maximum concentrations of lead and mercury in the Atrato River basin were 7.41 and 0.14 µg/g, respectively [19,20]. Hg levels in the air in Quibdó have reported with concentrations up to 24,610 ± 614 ng Hg/m^3^; likewise, Hg and Pb levels in the water of the Atrato River basin have presented values of 99.9 ± 37.4 and 0.003–0.159 ng/L, respectively [23]. Some research has been conducted on the effects of gold mining on some ecosystems associated with the Atrato river basin, including metal concentrations in fish and food as well as in humans, and risk assessments for human health have been performed [12,19,21,22,23,24,25,26]. The adverse effects of mining on the child population living in the riverside populations of the Atrato River basin have not yet been studied. For this reason, this research has the following main objective: (1) To carry out a socio-demographic characterization of the child population of the municipalities of Carmen de Atrato (CA), Río Quito (RQ), and Unguía (UN), emphasizing the economic activities of the parents, weekly consumption of fish, and handling of Hg amalgam. (2) To determine the levels of Hg and Pb in blood as a result of environmental exposure of the child population of the municipalities studied; (3) To establish the existing relationships between the concentrations of Hg and Pb in children with the socio-demographic characteristics of the study areas and the environmental problems generated by gold mining.

## 2. Materials and Methods

### 2.1. Study Area

**Figure 1:**
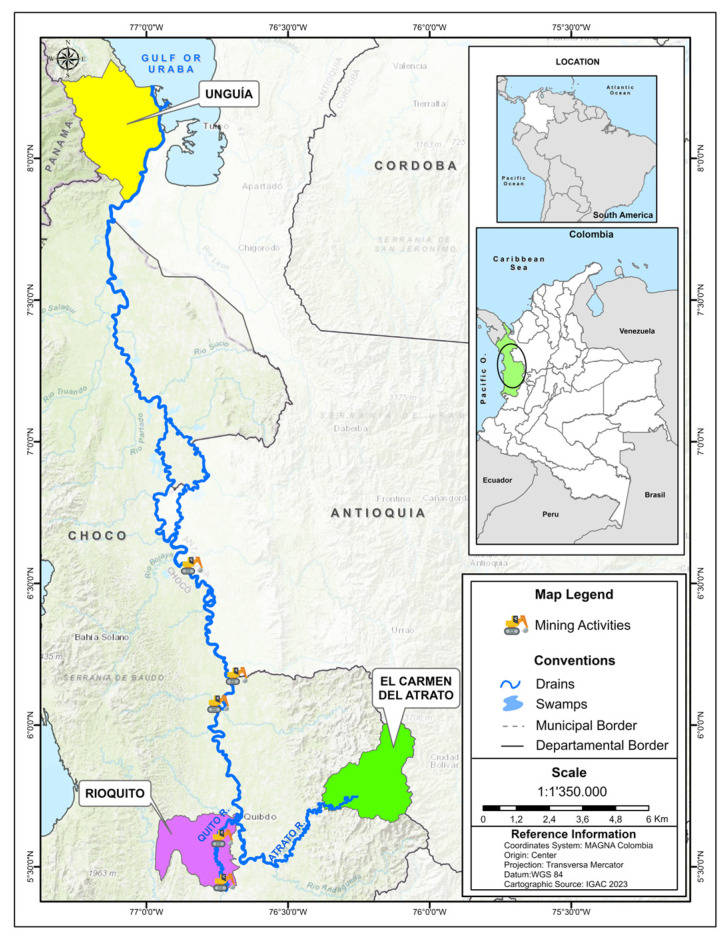
Map of the geographical location of the three municipalities influenced by the Atrato River basin (Chocó-Colombia): El Carmen Atrato, Río Quito, and Unguía.

This study was carried out in the municipalities of El Carmen de Atrato (CA), Río Quito (RQ), and Uguía (UN), belonging to the department of Chocó, which is located northwest of the Republic of Colombia (Figure 1). The sampling sites are located in the Atrato river basin, one of the main hydrographic basins in Colombia, highly contaminated and environmentally modified by gold extraction with Hg. The riverside communities of this basin base their economy and food security mainly on activities such as mining, fishing, and agriculture; however, mining is the main economic activity, which is developed based on an artisanal or small-scale manner. The use of machinery generates toxic waste such as heavy metals and loss of diversity in surrounding ecosystems as a result of obtaining gold with Hg. They were selected because they are strongly influenced by the waters of the Atrato River basin since the main economic activities in these municipalities are mining, fishing, and agriculture. In addition, they have an alarming lack of efficient garbage and solid waste collection services, and Río Quito has seen its main watershed destroyed by gold mining, creating a worrying social, environmental, food security, and health problem.

### 2.2. Sample Collection

Sociodemographic characteristics: In this study, the sociodemographic characteristics of the child population of the municipalities of RQ, CA, and UN were determined. A total of 171 children’s households were surveyed to determine aspects such as age, sex, level of education, geographic location, residence time, parents’ occupation, and frequency of fish consumption. The child population studied was between 5 and 14 years of age.

### 2.3. Blood Samples

Venous blood samples were taken from children accompanied by their parents, after explaining the procedures and signing the informed consent document. Briefly, 8 mL of blood was collected by venipuncture in test tubes containing ethylenediaminetetraacetic acid (EDTA). In the child population, a 25G x 1” f-gauge needle was used to collect the sample. The determinations were made using a direct Hg analyzer (DMA 80 Tri Cell) according to the United States Environmental Protection Agency 7473 method [27]. The samples obtained were refrigerated until their subsequent analysis. Subsequently, Pb levels were determined directly in whole blood using graphite furnace atomic absorption spectrometry with Zeeman background correction and a mixture of ammonium phosphate, Triton X-100, and HNO_3_ as matrix modifiers, according to the note of Method Guide app: 40,185 by Thermo Fisher Scientific Inc., Waltham, MA USA, (2008) [28].

The accuracy of the analytical procedure was verified through the analysis of certified reference materials RM IAEA-A-13 (Trace Elements in Freeze Dried Animal Blood). The variability of the results in the matrix was less than 7%. The results based on the recovery percentage were found within the acceptance interval reported by the Association of Official Agricultural Chemists (AOAC) (60–115%). The recovery percentage was 104.5%. The detection limit (LD) and quantification (LQ) were 0.05 μg/L and 0.166 μg/L for Hg and 0.06 μg/dL and 0.20 μg/dL for Pb, respectively.

### 2.4. Data Analysis

Kolmogorov–Smirnov (*n* ≥ 50) and Shapiro–Wilk (*n* < 50) tests were used to assess whether data followed a normal distribution. The Bonferroni test was employed to evaluate the differences between Hg and Pb concentrations among genders, municipalities, and ages. Spearman’s tests were performed to evaluate the correlation between the concentration of the elements and fish consumption. Correlation analyses between Pb and total Hg concentrations with gender and total fish intake were performed in the studied populations. A *p*-value of 0.05 was chosen to indicate statistical significance. Hg and Pb concentrations were expressed as μg/L and μg/dL of blood, respectively. The statistical analyses were carried out using the GraphPad Prism statistical program version 8.0.

### 2.5. Ethical Considerations

The Declaration of Helsinki and Resolution 8430 of 1993 by the Ministry of Health, which establishes the academic, technical and administrative standards for health research, classifies this research as minimal risk in Title II Chapter I, Article 11 on the ethical aspects of research in human beings. Therefore, for this study, signed informed consent forms were requested from each of the adults and/or legal representatives of the minor participants before answering the survey questions and taking biological samples.

## 3. Results and Discussion

Table 1 shows the sociodemographic variables of the children studied in the municipalities of CA, RQ, and UN belonging to the Atrato river basin. In general, 171 children were registered. In total, 52.63% *(n* = 90) of the evaluated population were boys, while 47.36% *(n* = 81) were girls. The population was grouped according to age, forming two groups of children 5–9 years old (*n* = 87; 50.88%) and 10–14 years old (*n* = 84; 49.12%), with a mean age of 9.8 years. In total, 88.90% of the child population evaluated were being educated.

**Figure 2:**
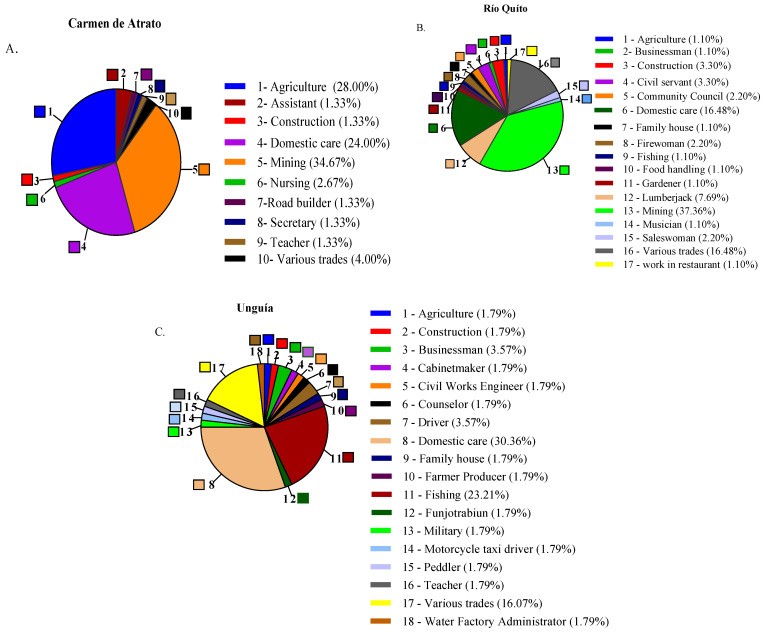
Occupation of the parents of the child population studied in the municipalities of (**A**) Carmen de Atrato, (**B**) Río Quito, and (**C**) Unguía.

The occupation of the parents showed that in the municipalities of CA and RQ, they focused on mining activity (35%, 37%), agriculture (28%, 16%), and domestic care (24%, 16%). In UN, most parents work in domestic care (23%), followed by fishing (16%) (Figure 2).

### Heavy Metal Concentrations in Children’s Blood

**Figure 3:**
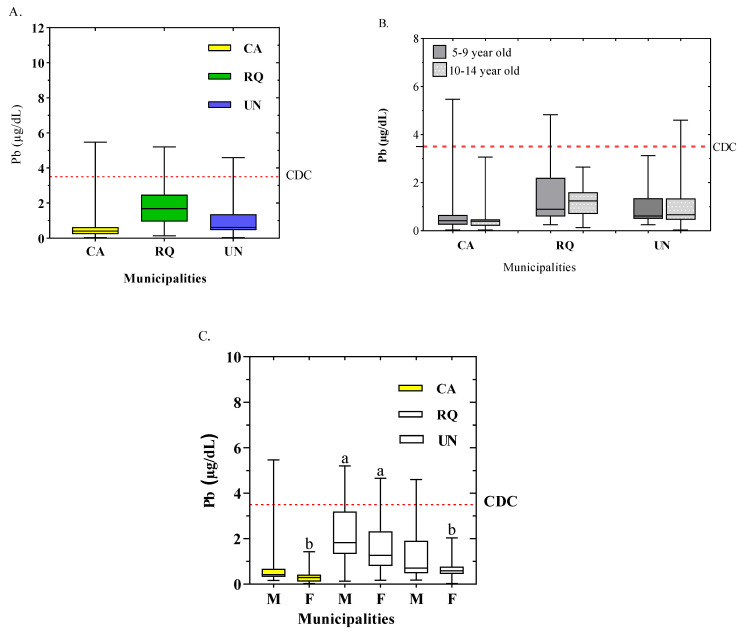
Box-and whisker-plots of blood Pb concentrations by municipality (**A**), Pb concentrations in blood in children in two age groups (5–9 and 10–14 years) by municipality (**B**), Pb concentrations in blood broken down by gender/sex in each municipality (**C**). a: above the reference value, b: below the reference value.

The concentrations of Pb in the child population of the Atrato River basin showed an average of 1.21 ± 1.20 μg/dL with ranges between 0.03 and 5.47 μg/dL (Table 2). Pb concentrations decrease in the following order: RQ > UN > CA (Figure 3A). In the study areas of the 171 children studied, 11 (18.8%) had blood Pb concentrations above the reference value established by the CDC (3.5 μg/dL). In general, for these populations, blood Pb concentrations were low.

Regarding the concentrations of Pb in blood in the municipality of CA (*n* = 51), this region presented an average of 0.59 ± 0.85 μg/dL and a range from 0.3 to 5.47 μg/dL. For this study area, it was found that 2% (1) of the child population had blood Pb concentrations above the reference value (3.5 μg/dL) (Figure 3A). In RQ (*n* = 64), the highest concentrations of Pb in blood were observed with a median of 1.85 ± 1.20 μg/dL and a range between 0.13 and 5.20 μg/dL. At this site, 11% (7) of the children evaluated presented concentrations above the reference value established by the CDC. In the municipality of UN (*n* = 56), Pb concentrations in blood showed an average of 1.04 ± 1.0 μg/dL and a range from 0.3 to 4.6 μg/dL. In total, 5.35% (3) of the children had blood Pb concentrations above the maximum allowed value (Figure 3A).

The average age of the child population under study in the Atrato River basin was 9.84 ± 2.86 years, with a range between 5.0 and 14.8 years. In general, children between 5 and 9 years of age presented an average of 1.44 ± 1.34 μg/dL, with a range of 0.03 to 5.47 μg/dL. On the other hand, children in the age range of 10–14 years presented a mean of 1.00 ± 0.90 μg/dL with values between 0.03 and 4.60 μg/dL (Figure 3B). The results showed that the child population was mainly composed of boys (*n* = 91), followed by girls (*n* = 80). In this study, 11% of the children had blood Pb concentrations between 3.60 and 5.47 μg/dL, which is above the threshold. Among girls, 1.25% had concentrations above the reference value (Figure 3C). In CA, 28 boys had concentrations above the reference value, with an median of 0.80 ± 1.08 μg/dL, ranging between 0.16 and 5.47 μg/dL; there were 23 girls with an median of 0.35 ± 0.33 μg/dL, ranging between 0.30 and 1.43 μg/dL (Figure 3C). In general, concentrations in boys were significantly higher than in girls in this municipality (*p* < 0.05), specifically between boys from RQ and girls from CA and UN. In general, Pb concentrations in boys are higher because of their behavior, they have the habit of being in contact with toys that have Pb, the ground, paints, or fishing equipment with weights, especially in these traditionally fishing areas [29]. Regarding the municipality of RQ, the values of Pb concentrations by gender did not present significant differences (*p* < 0.05). The children (*n* = 32) presented an average of 2.19 ± 1.33 μg/dL and a range of 0.13 to 5.20 μg/dL; girls (32) had an average of 1.52 ± 1.00 μg/dL and a range of 0.17 to 4.66 μg/dL (Figure 3C). Similarly, studies carried out in different places in the Colombian Caribbean showed blood lead levels of 3.5 ± 0.2 μg/dL in 554 children from 5 to 16 years old; Tasajera (Santa Marta-Colombia), a poor fishing community, reported an average of 8.9 ± 0.8 μg/dL [29] (Table 3). However, our results showed higher levels than reports of other studies related to exposure to blood lead levels associated with blood pressure in children and adolescents that reported concentrations between 0.46 and 0.96 µg/dL [11]. Likewise, reports in Spain showed that 155 children had increased Pb concentrations [30]. In China, among adolescents aged 12 years, the mean level of blood lead was 3.14 μg/dL [31]. In the cities of Landskrona and Trelleborg (Sweden), Pb concentrations between 0.23 and 5.9 µg/dL were reported [32]. In children living near a battery recycling plant in the cities of Zajača and Leskovac (Serbia), concentrations of 8.0 to 18.9 µg/dL were reported [33]. In Germany, in a study carried out on 270 children exposed to Pb due to domestic fuel and living with smokers at home despite not smoking themselves, average concentrations of 0.947 µg/dL were reported [34]. An average of 4.3 ± 0.75 µg/dL was reported in children under 6 years of age in Zimbabwe [35] (Table 3).

The high concentrations of Pb in the child population could be related to the following situations. First, due to the high mining activity, vegetation cover is lost, and the removal of a large amount of land causes the release of Pb contained in the soil [36], which generates a risk to the health of children, the population in general, and wildlife. There are studies that show that the levels of Pb in fruits and vegetables of high consumption in the middle basin of the Atrato River are above those established by the Codex Alimentarius of the FAO and WHO [26]. Likewise, in studies carried out in the city of Nabunturan (Philippines), considerably high levels of Pb were found in soil samples affected by gold mining [37]. Second, the use of poor-quality toys with high concentrations of Pb in the paint could contribute to the high levels of Pb in the child population given the precarious socioeconomic conditions in the study area; in fact, it is estimated that Chocó has a monetary poverty level of 68.4%, according to figures from National Administrative Department of Statistics [38]. In Nanjing, China, low-cost toys with high concentrations of Pb were found, posing a risk to children’s health [39,40]. Third, the fishing activity of parents along with the handling and availability of Pb in homes for use in the manufacturing of fishing nets could be another cause of high concentrations of Pb in children. It is important to note that the study areas have a fishing vocation, and fish consumption is the main source of protein for the population. There are reports of Pb concentrations in fish from the Atrato River basin exceeding the limits (*Ctenolucius beani*—1617.37 μg kg^−1^; *Cyphocharax magdalenae*—1111.27 μg kg^−1^; *Astyanax fasciatus*—427.85–407.69 μg kg^−1^) [25]. On the other hand, the results of this study agree with studies carried out in the towns of Canapote, Paseo Bolivar, Ceballos, Bosque, and Zaragocilla in the surroundings of an industrialized area of the city of Cartagena on the Colombian Caribbean coast, where the average concentrations were 1.7 µg/dL [41,42].

Fourth, the high concentrations of Pb in the blood of the child population could also be related to the parts of backhoes, dredgers, and batteries, among other tools that, after completing their cycle in the gold mining processes, are left abandoned in the communities.

**Figure 4:**
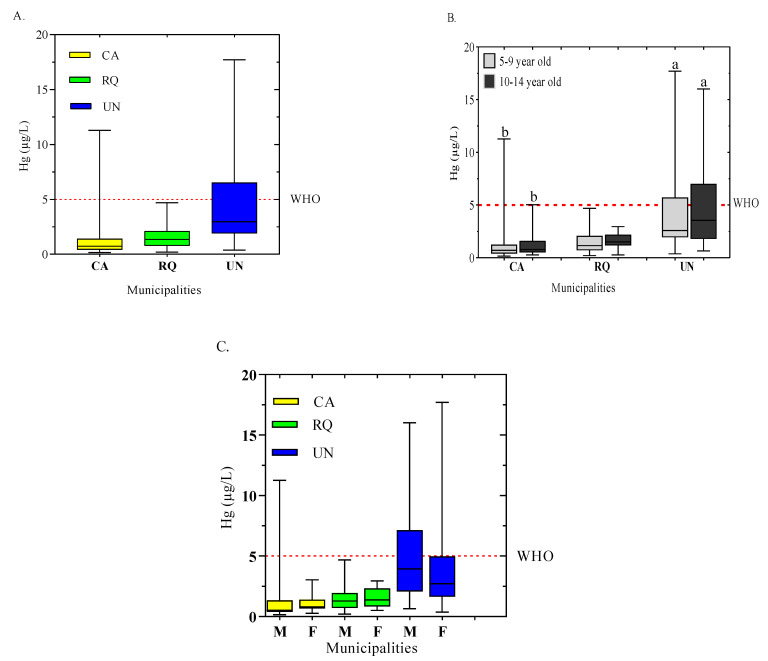
Box-and-whisker plots of blood Hg concentrations by municipality (**A**), Hg concentrations in blood in children in two age groups (5–9 and 10–14 years) by municipality (**B**), Hg concentrations in blood broken down by gender/sex in each municipality (**C**). a: above the reference value, b: below the reference value.

On the other hand, the lack of efficient garbage and solid waste collection systems in the municipalities studied, together with the poor disposal of electronic waste, cell phone batteries, and batteries, among others, aggravate the problems of Pb contamination because children could use this waste to play. This is because 50% of the municipalities near the Atrato River basin lack wastewater disposal and solid waste collection services [43]. This could be related to the reports of slightly higher levels in children aged 5–9 years reported in our study (Figure 4A,B). Similarly, in the municipality of UN, 31 children had an average of 1.34 ± 1.20 μg/dL, with a range of 0.20 to 4.60 μg/dL; girls (25) had an average of 0.67 ± 0.42 μg/dL, with a range of 0.03 to 2.04 μg/dL. No difference was found (*p* > 0.05) in the concentrations noted in children according to sex.

On the other hand, the child population in the Atrato basin reported average blood Hg concentrations of 2.50 ± 0.04 μg/L, with ranges between 0.16 and 17.71 μg/L. These concentrations decrease in the following order: UN > CA > RQ (Figure 4A). In the studied areas, 12.30% (21) of the children presented blood Hg concentrations above the threshold established (5 μg/L) by the World Health Organization [44]. In general, Hg concentrations for these populations were low. However, it was found that in UN and CA, 11.11% (19) and 1.17% (2) of the children evaluated presented blood Hg concentrations above the reference value, respectively. In the municipalities of UN (5.29–17.71 μg/L) and CA (5.03–8.43 μg/L) the maximum concentrations of Hg were observed, which are higher than the permitted level [45]. High concentrations of Hg in children could be related to the consumption of water contaminated by the atmospheric deposition of Hg on house roofs, crops, or the ground. Studies have shown that Hg travels long distances and can be deposited on the house roofs and be a contamination factor due to rainwater runoff. In Poland, concentrations of Hg were observed in the precipitation of the urban area of Poznań and at the Jeziory ecological station as rainwater samples were collected simultaneously at these sites [44,46]. Studies in children and adolescents in the Mojana region (Colombia) showed that 57.1% presented levels above the reference value according to the WHO for total mercury in hair and blood [19].

On the other hand, in areas such as the Atrato River basin, Hg is stored and used in the mining extraction process, forming gold amalgam. The gold is recovered by heating and volatilizing the Hg. The vapors generated represent another important source of contamination and a risk to the inhabitants, especially the children. Studies carried out in Madre de Dios (Perú) reported that amalgamation caused high concentrations in residents of an artisanal gold mining town, representing a health problem [47,48]. Similarly, our results are consistent with a study conducted in children (*n* = 88) aged 3–12 years old in a school and two kindergartens near mineral processing infrastructures in Mexico [49]. In mining areas such as the Atrato River basin, many people who handle toxic substances such as Hg do not report their use because their sale and use are prohibited.

CA, on average, presented concentrations of Hg in blood with a range that oscillated between 0.26 and 12.28 μg/L. In this area, it was found that 3.92% (2) of the child population (*n* = 51) presented concentrations of Hg in blood higher than the maximum level allowed. On the other hand, the municipality of RQ presented values that ranged from 0.20 to 4.7 μg/L. In this municipality, the entire population had blood Hg concentrations below the reference value established by the WHO. In the municipality of UN, the concentrations ranged from 0.37 to 17.71 μg/L. Of concern, the greatest adverse results were observed in this area, and 33.92% (19) of the child population (*n* = 51) presented concentrations of Hg in blood above that established by the WHO. The results showed that the highest values were recorded in children in age ranges of 5–9 years (Figure 4B), with values between 0.16 and 17.71 μg/L (median of 4.13 ± 4.00 μg/L); however, children between 10–14 years of age have a higher median (4.94 ± 4.10 μg/L). These concentrations were much higher than the reports of the blood Hg concentrations of children (0 to 7-year-old) in Shanghai, which ranged from 0.01 to 17.20 μg/L, with a median concentration of 1.34 μg/L [50] (Table 3).

Children in the age range of 10–14 years had concentrations between 0.27 and 16.03 μg/L, which represented 49.12% of the child population studied (Figure 4B). In addition, a significant difference (*p* <0.05) was found between these age ranges.

The reports from municipalities showed that in CA, the concentrations of Hg in blood in the child population from 5–9 years and 10–14 years exhibited values between 0.16 and 11.28 and 0.2 and 5.0 μg/L, respectively (Figure 4B). Similarly, in the municipality of RQ, children (5–9 years and 10–14 years) presented values that ranged from 0.20 to 4.70 μg/L and 0.30 to 2.95 μg/L, respectively. In terms of age, no difference in Hg concentrations was found (*p* > 0.05). In UN, children in the age range of 5–9 years had lower mean blood Hg concentrations (4.13 ± 4.00 μg/L) than children between 10–14 years, which ranged from 0.66 to 16.03 µg/L. However, there was no significant difference (*p* > 0.05) between Hg concentrations according to age. Regarding gender, boys (91) presented Hg concentrations between 0.16 and 16.03 μg/L, while girls (80) exhibited values between 0.27 and 17.71 μg/L. In general, 8.77% of boys and 3.51% of girls had blood Hg concentrations above the reference value (Figure 4C); however, there was no significant difference (*p* > 0.05) between blood Hg concentrations between boys and girls. These results are similar to recent reports of blood Hg concentrations in children 8–11 years old (0.01 to 17.20 μg/L) in the cities of Landskrona and Trelleborg (Sweden) [32]. In addition, studies carried out in China in children between 0 and 17 years of age observed Hg concentrations in blood that ranged between 0.01 and 17.2 μg/L [50]. However, in Germany, children aged 8–17 years reported lower concentrations (0.51 to 12.9 μg/L) [11] (Table 3).

In the municipality of CA, boys (28) and girls (23) reported blood Hg concentrations between 0.16 and 11.28 μg/L and 27 and 3.04 μg/L, respectively. However, only 3.92% of the children presented concentrations above the reference value without significant differences (*p* > 0.05). In RQ, none of the concentrations in either gender exceeded the limits (0.20 to 4.69 μg/L and 0.51 to 2.95 μg/L, respectively). This finding is contrary to the results in the municipality of UN, where concentrations in boys and girls were between 0.66 and 16.03 μg/L and 0.37 and17.71 μg/L, respectively, with 23.21% and 10.71% exceeding the reference value, respectively (Figure 4C).

**Figure 5:**
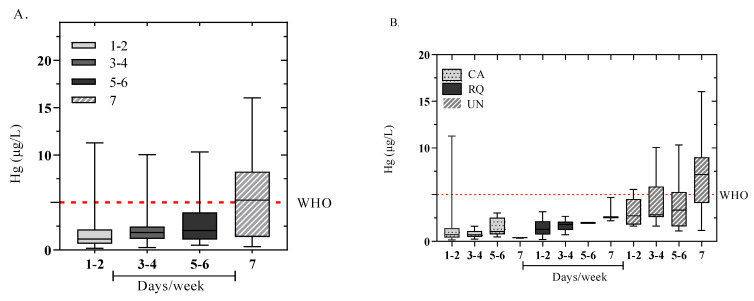
Box and whisker plots of the relationship between Hg concentrations in children’s blood and the frequency of consumption (day/week) of fish overall (**A**) and by municipalities (**B**).

According to the results obtained in this investigation, the concentrations of Hg in the blood of the child population were analyzed taking into account the frequency of consumption (day/week) of fish (Figure 5A). The frequency of consumption was divided into the following ranges: 1–2, 3–4, 5–6, and 7 days/week. Overall, the children (*n* = 63) who consumed fish 1–2 days/week reported a range of concentrations from 0.16 to 11.28. The children who consumed fish 3–4 days/week reported a range between 0.24 and 10.05 μg/L (*n* = 56). Those who ate fish 5–6 days/week had values that ranged from 0.48 to 10.33 μg/L (13), and those who ate fish 7 times a week had values that ranged from 0.34 to 16.03 μg/L (*n* = 20).

In CA, reports indicate that children (*n* = 27) who ate fish 1–2 times a week recorded maximum values of 11.28 μg/L, followed by children (n = 9) who ate fish 5–6 times a week with 3.04 μg/L, and finally children (*n* = 4) who ate fish 7 times a week with blood concentrations of 0.46 μg/L (Figure 5B). Similarly, in the municipality of RQ, children (*n* = 3) who ate fish 7 times a week had the highest concentrations at 4.69 μg/L, followed by children (*n* = 28) who ate fish 1–2 times a week with values of 3.18 μg/L (Figure 5B). Regarding the municipality of UN, children (3) who had the highest consumption of fish per week (7 times) showed the highest concentrations at 16.03 μg/L. In contrast, children (*n* = 8) who consumed fish only 1–2 days/week exhibited the lowest concentrations (range 1.64 to 5.55 μg/L). In mining areas such as the Atrato River basin, the consumption of fish contaminated with Hg may also be another important factor in the high levels of Hg in the communities since it is the main source of animal protein and sustenance in riverside populations settled in the Atrato River basin, contributing significantly to the food security of the communities. This has been evidenced in the frequency of fish consumption in the studied sites, where the municipality of UN presented the highest frequencies of consumption (Table 1, Figure 5B). This could explain the higher number of children aged 5–9 and 10–14 years with blood Hg levels higher than those allowed by the WHO in this municipality. These results are supported by research conducted in the Atrato River basin in 2020, 2022, and 2023, where more than 36.9% of highly consumed fish species exceeded the WHO limits. Furthermore, trophic level and habitat is important, and carnivorous and omnivorous fish have the highest levels. These reports showed total mercury concentrations in fish ranging from 32 ± 53 μg kg^−1^ (*Cyphocharax magdalenae*) to 678.5 ± 345 μg kg^−1^ (*Agneiosus pardalis*) (*n* = 842). In addition, values of 4.51 ± 5.75 μg kg^−1^ (*Oreochromis massabicus*) and 688.85 ± 332.24 μg kg^−1^ (*Agneiosus pardalis*) (*n* = 1372) and 32.9 ± 4.3 μg kg^−1^ (*Andinoacara pulcher*) and 1008.0 ± 552.7 μg kg^−1^(*Ctenolucius beani)* (*n* = 440) were also reported [24,25,26]. In addition, these results are consistent with research carried out in La Mojana (Colombia), where the inhabitants with higher concentrations of Hg had a higher frequency of fish consumption [51,52]. On the other hand, these results are higher than reports of children from other countries such as the USA [11], China [31], Sweden [32], France [53], and Spain [54] (Table 3).

It is important to clarify that these Hg concentrations in the blood of the child population evaluated in the different municipalities of the Atrato River basin reflect recent exposure to Hg; it is estimated that the half-life of Hg in the body is approximately 60 days [55]. This indicates that the child population in the Atrato River basin has been constantly exposed to Hg throughout their lives.

**Figure 6:**
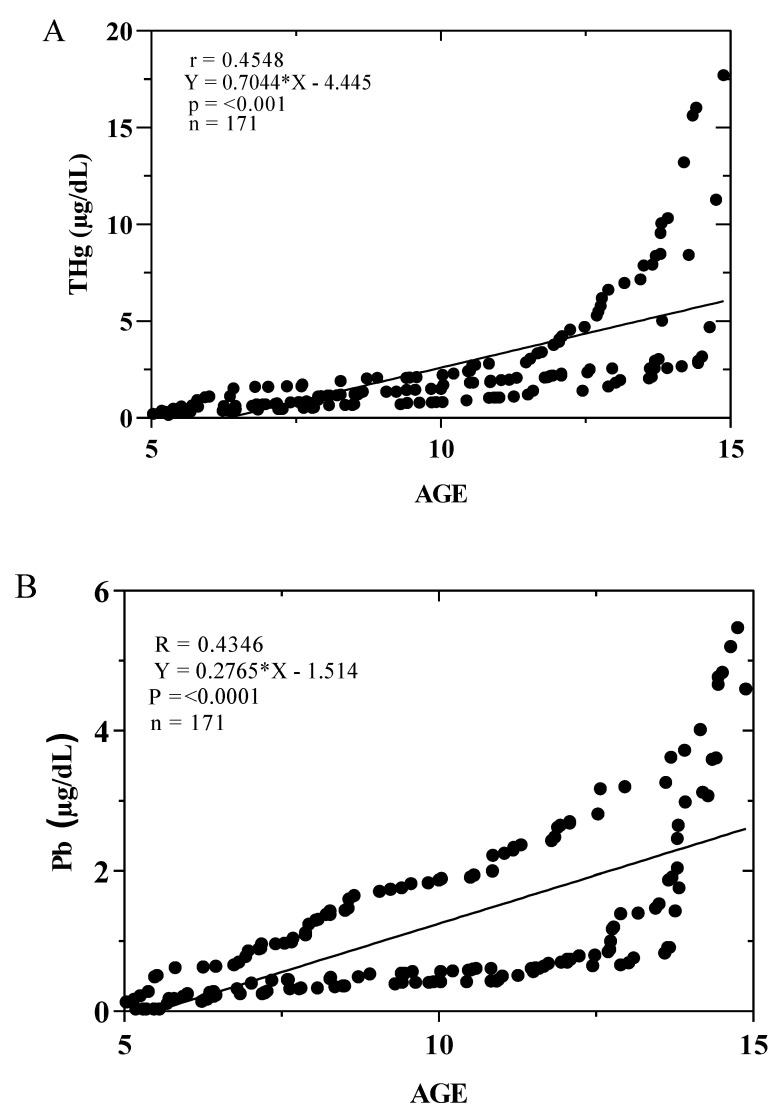
Linear regression analysis between the concentrations of Pb (**A**) and Hg (**B**) in the blood of the child population (μg/dL) of the Atrato River basin and their chronological ages.

In general, when the results of the three municipalities were grouped by age, the concentrations of Hg in blood were more significant than those of Pb. It was observed that the municipality of UN presented the highest concentrations of Hg in blood both in children between 5–9 and 10–14 years of age, and the mean of these concentrations did not exceed the threshold. However, there were children who exceeded the established limit. On the other hand, when grouping the results of the municipalities by sex, the average concentrations of Pb were low; however, children from the municipality of RQ showed concentrations close to the threshold. On the other hand, the linear regression analyses suggest that there is a linear relationship between the concentrations of Pb and Hg in the blood of the child population of the Atrato River basin and the age of the children studied (Figure 6).

**Table 3:** Chronologically organized summary of studies (2017–2024) on THg (µg/L) and Pb (µg/dL) in blood of children collected at various worldwide locations.

Location	Mean THg	Mean Pb	Range	*n*	Possible Pollution Sources	Remarks	Reference
Cartagena, Colombia	-	1.7	0.05–34.05	118	Urban sewage waters and industrial sewage	Cartagena Bay and the Tesca Marsh, two water bodies with inputs from urban sewage waters from Cartagena. Its industrial zone is located around the bay, and the main industries include petrochemical-derived activities, oil refining, pesticide manufacturing, and food processing.	[29]
Zajača and Paskovac, Serbia	-	12	8.0–18.9	127	Battery recycling	Children living near a battery recycling plant in Serbia.	[32]
Santa Marta, Valledupar, Tasajera, La Paz Cartagena, Colombia	-	3.5	0.1–50.1	554	Fishing community	Manipulation and use of Pb in this place, not only by adults, who employ it for manufacturing fishing nets, but also by children.	[41]
USA	0.44	0.67	0.46–0.96 (Pb) 0.23–0.70 (THg)	7076	Study using data from the National Health and Nutritional Examination Surveys (NHANES)	Children and adolescents with blood lead and Hg levels associated with blood pressure. The participants included Mexican Americans, other Hispanics, whites, blacks, and other races.	[11]
Spain	-	1.1	0.7–1.6	155	Sociodemographic variables; daily habits; characteristics of the subject’s current Residence; parental smoking; possible occupational exposure of one or both parents in the past or present; hobbies of either of the parents such as artistic painting, furniture restoration or ceramics; parents’ highest level of education; type of drinking water; use of cooking utensils, etc.	Study was performed at the Trace Element Department of Hospital Clínico San Carlos in Madrid (Spain) in 155 children.	[30]
Germany	-	0.947	0.51–12.9	720	Participants with domestic fuel and living with smokers in the household but not smoking themselves	In the German Environmental Survey of 2014–2017, 720 samples from participants aged 3 to 17 years of the German Health Interview and Examination Survey for Children and Adolescents of the Robert Koch Institute were examined.	[34]
China	1.26	3.14	2.3–3.7 (Pb) 0.7–1.7 (THg)	532	Air pollution resulting from factory emissions	The association between low levels of environmental lead, Hg and resting heart rate was examined in 532 adolescents aged 12 years, in China.	[31]
Changai, China	1.34	-	0.01–17.2	1474	Higher frequency of consuming aquatic products, rice, vegetables, and formula milk were identified as risk factors.	This study measured blood Hg levels and identified potential factors influencing blood Hg levels, including demographic and socioeconomic factors, lifestyle, and daily dietary habits, among 0- to 7-year-old children in Shanghai.	[49]
Harare, Zimbabwe		4.3	-	86	Old houses painted with lead paint	This study measured blood lead levels in 86 children living in Mbare, a densely populated suburb in Harare, Zimbabwe, characterized by dwellings progressively constructed from 1907 to the 1940s, before the ban of lead paint.	[35]
Landskrona and Trelleborg, Sweden	0.73	0.99	0.23–5.9 (Pb) 0.02–8.2 (THg)	773	Pb smelter, the biggest recyclers of used Pb-acid batteries in Europe, which recovers Pb from around four million used car batteries per year	This study follows temporal changes in blood lead and mercury levels in schoolchildren (8–11 years old) from two cities in southern Sweden, and 773 children were included in the analyses.	[32]
District of Goslar, Bad Harzburg, Germany		188.9	80–658	75	Exposure to secondhand smoke at home, length of residence at current address, soil with high lead levels	Biomonitoring study among children living in two former mining communities in Lower Saxony, Germany. In these communities, the soil contains lead levels of 1000 to 30,000 mg/kg.	[56]
Guadeloupe (French West Indies), French	1.7	220	162–271 (Pb) 1.0–2.8 (THg)	310	Prenatal and childhood exposure to pesticides and other environmental pollutants (including heavy metals) for pregnancy and child development	In Guadeloupe, an island in the Caribbean Sea, inhabitants regularly eat large quantities of seafood (estimated at 35–37 kg/habitant/year), mostly smaller predatory fishes such as snapper, dolphinfish, catfish, grouper, or tuna, which are moderately contaminated by methylmercury.	[53]
Sabadell (Catalonia), Spain	5.8	-	-	151	Exposure to tobacco smoke; secondhand smoke exposure at home; diet; intake of fish, fruits, and vegetables	Contribution of exposure to tobacco and mercury from the prenatal period to childhood to individual differences in the fecal microbiome composition of 7-year-old children.	[54]
Boston, MA, USA	-	1.0	0.14–6.9	349	Exposure maternal first-trimester erythrocyte metal concentrations; diet	In Boston, the associations of a panel of 15 non-essential and essential metals measured in early childhood erythrocytes with child cognitive test scores obtained in early and mid-childhood (visual-motor ability, language, memory, and general intelligence) were estimated.	[57]
El Carmen de Atrato, Río Quito, and Uguía, Colombia	2.5	1.21	0.03–5.47 (Pb) 0.16–17.71 (THg)	171	Consumption of fish contaminated with heavy metals Toxic waste generated by small-scale gold mining	The sampling sites are located in the Atrato River basin, one of the main hydrographic basins in Colombia, highly contaminated and environmentally modified by gold extraction with Hg.	This Study

**Figure 7:**
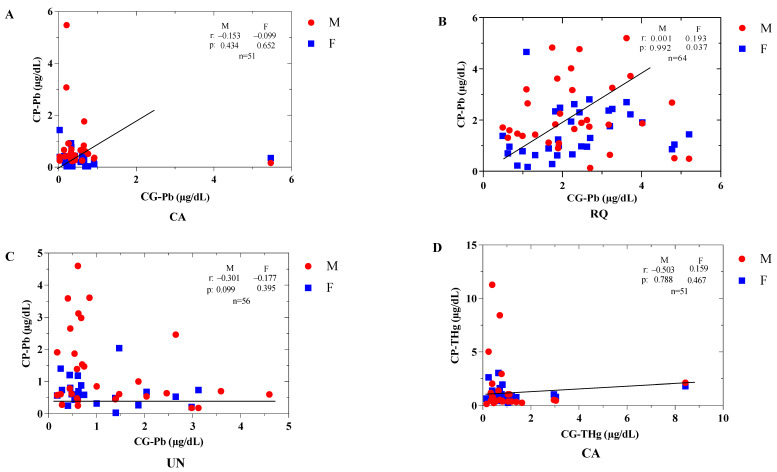

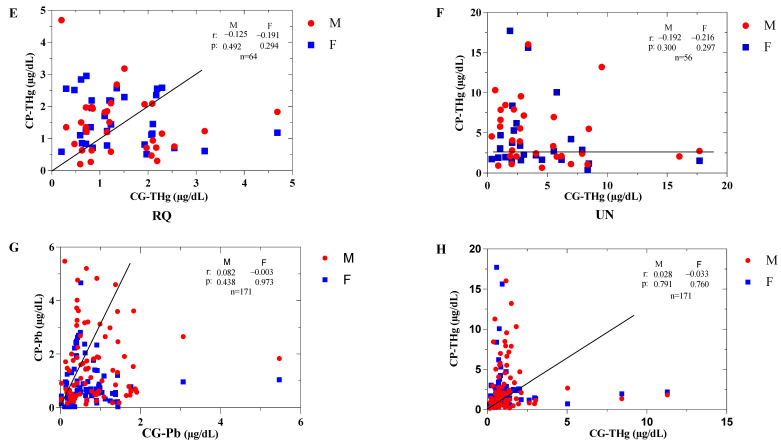

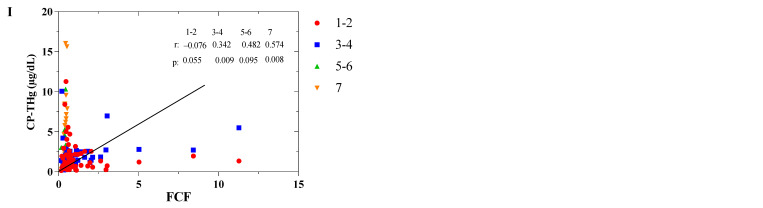
Pearson correlation analysis between (**A**) total concentrations of Pb in blood in the population (μg/dL) and total concentrations of Pb by gender (μg/L) in CA, (**B**) RQ, and (**C**) UN; (**D**) total concentrations of Hg in blood in the child population (μg/dL) and total concentrations of Hg by gender in CA, (**E**) RQ and (**F**) UN; (**G**) total concentrations of Pb in blood in the child population (μg/dL) and total concentrations of Pb by gender (μg/L) in the municipalities; (**H**) total concentrations of Hg in blood in the child population (μg/dL) and total concentrations of Hg by gender (μg/L) in the municipalities; (**I**) total concentrations of Hg in the population (μg/dL) and frequency of fish consumption (days/week). CP-Pb: total concentrations of Pb in the population (μg/dL); CG-Pb: total concentrations of Pb by gender (μg/dL); CP-THg: total concentrations of Hg in the population (μg/dL); CG-THg: total concentrations of Hg by gender (μg/dL); FCF: frequency of fish consumption (days/week).

The Pearson correlation analysis between the total concentrations of Pb and Hg in the population based on gender and the frequency of fish consumption demonstrated few significant relationships. A low positive correlation was observed between total Hg concentrations in the population (CP) and fish consumption 3–4 days per week (r: 0.342; *p* < 0.009), and a moderate positive correlation was noted for the consumption of 7 days per week (r: 0.574; *p* < 0.008) (Figure 7I). Furthermore, there is a moderate positive correlation, albeit not significant, for the consumption of fish for 5–6 days (r: 0.482; *p* < 0.095). Likewise, there is a significant moderately low positive correlation between CP-Pb and CG-Pb for the female gender in RQ (r: 0.193; *p* < 0.037) (Figure 7B). The other correlations were moderately low positive or negative without statistical significance (Figure 7).

## 4. Conclusions

For the first time, records of Pb and Hg concentrations in blood are reported in children in areas of the Atrato River basin, an area highly impacted by gold mining. These results provide information on the existing problem in the Atrato basin due to the heavy metals evaluated, which are reaching children, revealing anthropogenic activities such as mining, agriculture, and fishing, as well as precarious or non-existent access to drinking water and basic sanitation.

The concentrations of Hg and Pb in blood registered a similar distribution with respect to the variables sex and age of the child population; boys generally reported higher concentrations than girls. Regarding the average concentrations of Pb and Hg in children’s blood, there were significant differences between the municipalities of RQ and UN.

In the study areas of the 171 children studied, overall, 11 children had blood Pb concentrations above the reference value established by the CDC (3.5 μg/dL), including one child in CA, seven in RQ, and three in UN. The concentrations of Hg in the blood of children in this study were also above the WHO standard (5.0 μg/dL), but only 18 in CA and three in UN presented these reports.

It was recorded that children with a higher frequency of fish consumption presented higher concentrations of Hg in the blood, revealing a significant difference (*p* < 0.05) between the evaluated municipalities. These findings indicate that fish is a source of exposure to Hg for populations settled in the Atrato River basin.

## Figures and Tables

**Table 1:** General characteristics of the child population studied (5–14 years) in the municipalities of the Atrato river basin, Colombia (*n* = 171).

Characteristic	Total	CA	RQ	UN
Age, *n* (%)	171	51	64	56
5–9 years	87	30 (59)	37 (58)	20 (35)
10–14 years	84	21 (41)	27 (42%)	36 (65)
Gender, *n* (%)				
Male	90	28 (55)	32 (50)	31 (55)
Female	81	23 (45)	32 (50)	25 (45)
Amalgam cremation at home				
Yes	22	7 (14)	15 (23)	0 (0)
No	149	44 (86)	49 (77)	56 (100)
Stores Hg, *n* (%)				
Yes	20	7 (14)	13 (20)	0 (0)
No	151	44 (86)	53 (80)	54 (100)
Eat fish				
Yes	158	46 (90)	54 (84)	55 (98)
No	13	5 (10)	9 (16)	1 (2)
Fish consumption (days/week), *n* (%)				
1–2	65	28 (60)	29 (58)	8 (20)
3–4	38	9 (20)	19 (38)	10 (26)
5–6	13	5 (11)	1 (2)	7 (18)
7	19	4 (9)	1(2)	14 (36)
Study, *n* (%)				
Yes	152	41 (79)	57 (97)	54 (96)
Not	15	11 (21)	2 (3)	2 (4)

CA: El Carmen de Atrato; RQ: Río Quito; UN: Unguía.

**Table 2:** Distribution of blood Pb concentrations (median ± standard deviation, μg/dL) in the children studied in the different municipalities according to sex and age.

Population Characteristic	*n* (%)	Pb, Hg Concentrations in CA (μg/dL)		*n* (%)	Pb, Hg Concentrations in RQ (μg/dL)		*n* (%)	Pb, Hg Concentrations in UN (μg/dL)	
Age; Sex	51	0.60 ± 0.85	1.40 ± 2.00	64	1.85 ± 1.20	1.51 ± 0.90	56	1.04 ± 1.00	4.65 ± 4.03
5–9 years	30 (59)	0.63 ± 1.00	1.509 ± 2.41	37 (58)	2.35 ± 1.30	1.51 ± 1.00	20 (36)	1.00 ± 0.81	4.13 ± 4.00
10–14 years	21(41)	0.53 ± 0.70	1.20 ± 1.10	27 (42)	1.20 ± 0.65	1.55 ± 0.63	36 (64)	1.10 ± 1.10	4.94 ± 4.10
M	28 (55)	0.80 ± 1.10	1.60 ± 2.60	32 (50)	2.20 ± 1.33	1.50 ± 0.80	31 (55)	1.34 ± 1.20	4.80 ± 3.80
F	23 (45)	0.35 ± 0.33	1.10 ± 0.71	32 (50)	1.52 ± 1.00	1.60 ± 0.92	25 (45)	0.70 ± 0.42	4.31 ± 4.40

CA: El Carmen de Atrato; RQ: Río Quito; UN: Unguía; %: percentage of samples; n: sample size; M: male; F: female.

## Data Availability

All data are included in the main text.
